# A single session of aerobic exercise reduces systolic blood pressure at rest and in response to stress in women with rheumatoid arthritis and hypertension

**DOI:** 10.1038/s41371-023-00869-z

**Published:** 2023-10-19

**Authors:** Tatiane Almeida de Luna, Diego Augusto Nunes Rezende, Leandro Campos de Brito, Rafael Yokoyama Fecchio, Fernanda Rodrigues Lima, Ana Lúcia de Sá Pinto, Ana Cristina de Medeiros Ribeiro, Karina Rossi Bonfiglioli, Bruno Gualano, Hamilton Roschel, Tiago Peçanha

**Affiliations:** 1 Applied Physiology and Nutrition Research Group, School of Physical Education and Sport and Faculdade de Medicina FMUSP, Universidade de Sao Paulo, Sao Paulo, SP Brazil; 2 Laboratory of Assessment and Conditioning in Rheumatology, Hospital das Clínicas HCFMUSP, Faculdade de Medicina FMUSP, Universidade de Sao Paulo, Sao Paulo, SP Brazil; 3 Exercise Hemodynamic Laboratory, School of Physical Education and Sport, University of São Paulo, Sao Paulo, SP Brazil; 4 Applied Chronobiology and Exercise Physiology, School of Arts, Sciences and Humanities, University of São Paulo, Sao Paulo, SP Brazil; 5 Oregon Institute of Occupational Health Sciences, Oregon Health & Science University, Portland, OR USA; 6 Rheumatology Division, Hospital das Clínicas HCFMUSP, Faculdade de Medicina FMUSP, Universidade de Sao Paulo, Sao Paulo, SP Brazil; 7 Department of Sport and Exercise Sciences, Manchester Metropolitan University Institute of Sport, Manchester Metropolitan University, Manchester, UK

**Keywords:** Hypertension, Medical research

## Abstract

Rheumatoid arthritis (RA) is an autoimmune inflammatory disease characterized by increased risk of cardiovascular disease and hypertension (HT). A single session of aerobic exercise may reduce blood pressure (BP) in different clinical groups; however, little is known about the acute effects of exercise on BP in RA patients. This is a randomized controlled crossover study that assessed the effects of a single session of aerobic exercise on resting BP, on BP responses to stressful stimuli, and on 24-h BP in women with RA and HT. Twenty women with RA and HT (53 ± 10 years) undertook sessions of 30-min treadmill exercise (50% VO_2max_) or control (no exercise) in a crossover fashion. Before and after the sessions, BP was measured at rest, and in response to the Stroop-Color Word Test (SCWT), the Cold Pressor Test (CPT), and an isometric handgrip test. After the sessions, participants were also fitted with an ambulatory BP monitor for the assessment of 24-h BP. A single session of exercise reduced resting systolic BP (SBP) (−5 ± 9 mmHg; *p* < 0.05), and reduced SBP response to the SCWT (−7 ± 14 mmHg; *p* < 0.05), and to the CPT (−5 ± 11 mmHg; *p* < 0.05). Exercise did not reduce resting diastolic BP (DBP), BP responses to the isometric handgrip test or 24-h BP. In conclusion, a single session of aerobic exercise reduced SBP at rest and in response to stressful stimuli in hypertensive women with RA. These results support the use of exercise as a strategy for controlling HT and, hence, reducing cardiovascular risk in women with RA.

**Clinical Trial Registration:** This study registered at the Brazilian Clinical Trials (https://ensaiosclinicos.gov.br/rg/RBR-867k9g) at 12/13/2019.

## Introduction

Rheumatoid arthritis (RA) is a chronic autoimmune inflammatory disease affecting synovial joints and causing pain, swelling and progressive physical incapacity [1]. RA is also characterized by increased rates of cardiovascular disease (CVD) morbidity and mortality [2], which has been attributed to a complex interplay between traditional risk factors and the direct effects of sustained inflammation upon the heart and the vasculature [3, 4].

Hypertension is a well-established risk factor for the development of CVD in RA [5–7]. Hypertensive RA patients have a 2-fold increased risk of cardiovascular morbidity compared with non-hypertensive RA patients [5]. Of relevance, patients with RA also present higher rates of hypertension [6, 8], lower rates of hypertension control [6, 8] and higher blood pressure lability than the general population [9]. Poor hypertension control has been associated with increased cardiovascular risk in this population [7, 9]. This reinforces the importance of non-pharmacological approaches focused on improving hypertension management in RA patients.

A single session of aerobic exercise has been shown to promote a transient reduction in BP below resting values, which is termed as post-exercise hypotension (PEH) [10]. PEH has been defined as a phenomenon of clinical relevance as it has significant magnitude [11], and may last for many hours [12], offering to individuals with hypertension the benefit of transiently reducing their BP for a significant part of the day. Additionally, exercise reduces BP not only at rest or in ambulatory conditions, but it also may reduce the BP reactivity to stressful stimulation, such as during mental or physical stress [13], which may also contribute to reducing the risks of acute cardiovascular events in this population.

Despite compelling evidence showing the beneficial effects of exercise on BP control in different clinical populations, there is still scarce information on the acute effects of exercise on BP in RA. Specifically, it is unknown if patients with RA would experience PEH as previous studies have demonstrated altered cardiovascular responses to exercise and to other relevant physiological stimuli in this population [14, 15]. It is also unknown if exercise can reduce BP reactivity to stressful stimuli and in ambulatory conditions in RA. Therefore, the aim of this study was to assess the effects of a single session of moderate-intensity aerobic exercise on resting BP, on BP responses to stressful stimuli and during ambulatory conditions in hypertensive RA women.

## Materials and methods

### Participants

The sample consisted of 20 women with RA [16] from the Rheumatoid Arthritis clinic of the Clinics Hospital of the School of Medicine of the University of Sao Paulo. Potential participants were identified from the clinic data records and all of those who complied to the study eligibility criteria (detailed below) were invited to participate in the study via a phone call. To participate in the study, the participants should have a diagnosis of RA according to the American College of Rheumatology criteria [16], and be on a stable drug therapy in the last 3 months and throughout the study. All participants should also be hypertensive, which was defined according to established criteria [17]. Exclusion criteria were as follows: a body mass index (BMI) ≥ 35 kg/m^2^; presence of heart, pulmonary or renal diseases, stroke, diabetes, and severe musculoskeletal and joint disorders that could preclude the execution of the tests; use of medications that could affect cardiovascular responses to exercise; abnormal resting or exercise electrocardiograms.

Prior to participation in the study, participants received a detailed explanation about the experimental procedures and provided their written informed consent. The study followed the principles of the Declaration of Helsinki and was approved by the local Institutional Ethics Committee (Clinics Hospital, University of Sao Paulo. CAAE: 90250718.0.0000.0068).

### Study design

This was a randomized crossover trial (trial registration: https://ensaiosclinicos.gov.br/rg/RBR-867k9g). Participants attended the laboratory on four separate occasions. In the first two visits, participants were screened to determine eligibility, signed informed consent form, and performed the baseline tests. In the third and fourth visits, participants performed two experimental sessions (exercise and control), which were conducted in a random manner (simple randomization using https://www.randomizer.org/), with an interval of at least 48 h between them.

### Baseline tests (visits one and two)

#### Clinical evaluation

Demographic and clinical data (disease duration, disease activity, drug therapy, co-morbidities) were obtained through personal interview and medical records. Disease activity was determined by means of the Disease Activity Score-28 with C-reactive protein (DAS28-CRP) [18], whereas functional capacity was assessed through the Health Assessment Questionnaire (HAQ) [19]. Participant’s current pain level was assessed by a 0–10 visual analogue scale (Pain-VAS). Participants were also questioned about their physical activity level and the amount of weekly leisure time physical activity and were defined as active if they performed at least 150 min per week of moderate-to-vigorous physical activity [18] or insufficiently active if they did not reach that threshold.

Blood pressure (BP) was measured in two different days (visits 1 and 2), by an automated oscillometric device (Mindray MEC-1000, Shenzhen Mindray Bio-Medical Electronics Co, China) after 10-min seated rest, following the recommendations of the Brazilian Guidelines of Hypertension [19]. Briefly, BP was measured three times on both arms on visit 1 and then BP was measured three additional times on the arm with highest BP on visit 2. Baseline BP was defined as the mean of six measures on the arm with the highest BP across visits 1 and 2.

Blood samples (30 ml) were collected after a 12-h overnight fast for characterization of the cardiometabolic profile of the participants. We assessed glucose, lipid profile (i.e., high-density lipoprotein (HDL) cholesterol, low-density lipoprotein (LDL) cholesterol, very low-density lipoprotein (VLDL) cholesterol, total cholesterol, and triglycerides), C-reactive protein (CRP) and erythrocyte sedimentation rate (ESR).

#### Cardiopulmonary exercise test

Participants performed a maximal cardiopulmonary exercise test (CPET) on a treadmill, following a stepwise protocol with increments of speed and incline every 3 min. Initial workload and workload increments were set according to participants’ perceived fitness estimated based on an initial familiarization with the treadmill ~1 h before the CPET. During the CPET, oxygen consumption (VO_2_) was collected breath-by-breath using a metabolic cart (Metalyzer III B/ breath-by-breath, Leipzig, Germany) and heart rate (HR) was measured using a 12-lead ECG. Peak VO_2_ (VO_2peak_) and HR (HR_peak_) were defined as their maximal values attained at the end of the exercise test (average of 30 s).

### Experimental sessions (visits 3 and 4)

Participants attended the laboratory in two separate occasions to perform the experimental sessions (exercise and control). These sessions were performed in a temperature-controlled laboratory (20–22 °C) during the afternoon. Participants were instructed to avoid the intake of caffeinated and alcoholic beverages and not to perform strenuous exercise in the 24 h preceding the sessions. Additionally, they were instructed to have a light meal 2 h before the sessions. To avoid the confounding effects of different eating habits before the experimental sessions, participants completed a 24-hour food recall prior to the first experimental session and were asked to repeat a similar diet prior to the second session. Women of reproductive age performed the sessions on the first 7 days of their menstrual cycle, or during the inactive/placebo phase of the oral contraceptive pack for oral contraceptive pill users.

**Fig. 1:**
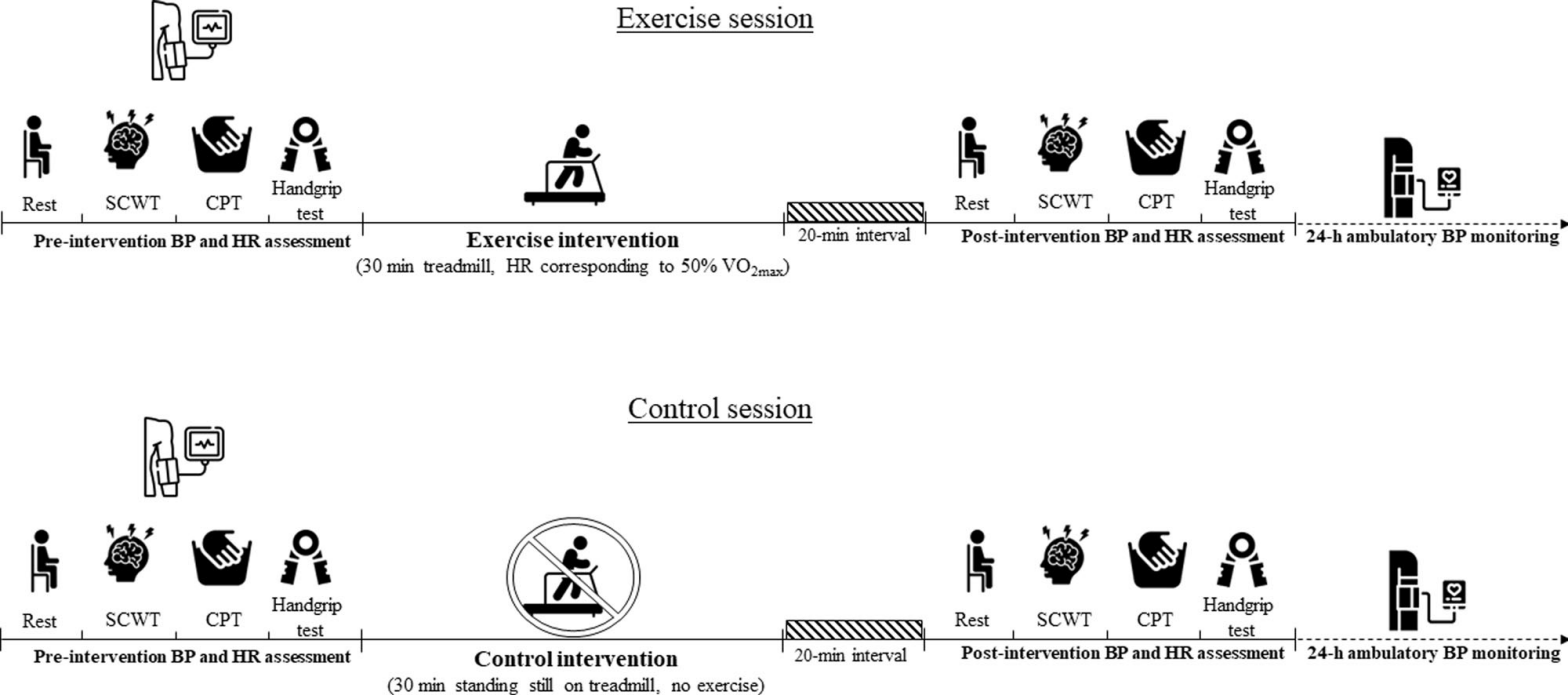
Summary of the experimental sessions. The exercise (upper panel) and control sessions (lower panel) were performed in different days and the order of the experiments was randomised. Both sessions were composed by pre- and post-interventions of blood pressure (BP) and heart rate (HR) at rest and in response to the Stroop Color and Word Test (SCWT), cold pressor test (CPT) and handgrip test. After the post-intervention assessments, the participants were fitted with an ambulatory BP monitor for 24-h BP assessment. The black and white icons were created by Freepik/Flaticon.

The experimental sessions were composed by (a) pre-intervention assessments of BP and HR at rest and in response to different stress; (b) intervention (exercise or control); (c) post-intervention assessments of BP and HR at rest and in response to different stress and; (d) 24-h (out of the lab) ambulatory BP assessment. Importantly, participants remained seated at rest for 20 min between the intervention and the post-intervention assessments. Figure 1 presents a summary with the timeline of the assessments and interventions performed in both sessions.

### Intervention

In the exercise session, participants performed a 30-min exercise on a treadmill at a heart rate (HR) corresponding to 50% VO_2max_. In the control session, participants remained stood in treadmill during 30 min, but without performing any exercise.

### Pre- and post-intervention assessments

Assessments were taken during the pre- and post-intervention periods with the subjects in the sitting position. BP was assessed in the left arm using an automated oscillometric device, and HR by a 3-lead ECG connected to a multiparameter monitor (Mindray MEC-1000, Shenzhen Mindray Bio-Medical Electronics Co, China). These assessments were performed at rest, and during the Stroop Color and Word Test (SCWT), the Cold Pressor Test (CPT) and the handgrip test (detailed below). A 3–5 min recovery period was provided between each test for the return of HR and BP to baseline.

#### Rest

Resting BP and HR were assessed after 10-min resting in the sitting position. BP and HR were measured three times, and resting BP and HR were considered the mean of all three measurements. PEH was calculated as the net effect of exercise on BP (i.e., the difference between responses observed in the exercise and control sessions) by the formula: PEH net effect = (post-exercise BP – pre-exercise BP) – (post-control BP – pre-control BP) [20, 21].

#### Strop color and word test

A modified version of the SCWT was used to promote mental stress in the study participants. The test started with 3-min baseline, and then the participants were presented with a sequence of words of different colors (red/green/blue) printed in either same (congruent condition) or different colors (incongruent condition). Participants were requested to respond the color of the ink rather than the words meaning [22]. The participants were asked to name each word color in the fastest possible way. At the end of the test, participants were asked to rate their perceived stress during the test on a 5 point scale (0–4, where 0 was ‘non-stressful’ and 4 was ‘extremely stressful’) [23]. Participants could only choose a whole number value (i.e., decimals were not allowed).

HR and BP were measured in 1-min intervals during the test, and SBP, DBP and HR reactivity to the SCWT were calculated as the absolute changes (delta changes – Δ) from mean baseline to their highest values achieved during the stress phase (usually within the 2nd of 3rd minute of stress).

#### Cold pressor test

The CPT is a reactivity test that evokes significant increases in BP and HR as a consequence of pain-induced sympathoexcitation [24]. The test started with 3-min baseline, and then the participants were instructed to immerse their right hand until the wrist in water at 4 °C for 3 min. At the end of the test, participants were asked to rate their pain on a 0-10 visual analogue pain scale (0 = not painful, 10 = maximal tolerable pain).

HR and BP were measured in 1-min intervals during the test, and SBP, DBP and HR reactivity to the CPT were calculated as the absolute changes (delta changes – Δ) from mean baseline to their highest values achieved during the hand immersion phase (usually within the 2nd of 3rd minute of hand immersion).

#### Handgrip test

The handgrip test was performed with a hand dynamometer (Crown Manual-50KGF, INSTRUTEMP, Sao Paulo, Brazil). The test consisted of 3 min baseline and then participants were asked to perform 3-min of sustained isometric contraction in the hand dynamometer at 30% of the maximal isometric voluntary contraction (MVC) using their right hand. During the test, participants received feedback from the investigators in order to sustain the targeted force. At the end of the test, participants were asked to rate their pain on a 0–10 visual analogue pain scale (0 = not painful, 10 = maximal tolerable pain).

HR and BP were measured in 1-min intervals during the test, and SBP, DBP and HR reactivity to the handgrip test were calculated as the absolute changes (delta changes – Δ) from baseline to their highest values achieved during the handgrip (usually within the 2nd of 3rd minute of the handgrip test).

#### 24-hours blood pressure

After the post-intervention testing (~17:00), participants underwent an ambulatory BP monitoring for 24-h. Measurements were taken every 15 min for 24 h (this same frequency of measurements were kept during sleep) using an oscillometric device (CardioMapa, CARDIOS, São Paulo, Brazil). Participants were instructed to complete a diary with time of sleeping, waking, medication use and daytime activities. On the completion of the test, data was downloaded to an offline PC using the software Dyna-MAPA (CARDIOS, Sao Paulo, Brazil), that calculated the mean of SBP, DBP and HR for the entire 24 h, and for the asleep and awake periods as reported by the patients in the diary. Additionally, SBP and DBP nocturnal fall (%) was calculated by the difference between asleep SBP/DBP and awake SBP/DBP, divided by awake SBP/DBP and multiplied by 100. Only exams with more than 80% of measurements were considered valid for analysis [25].

### Statistical analysis

Normality of data and homogeneity of variance in each group were checked with the Shapiro-Wilk and the Levene tests, respectively. A two-way (session vs. time) repeated measures analysis of variance was used to compare resting BP and BP reactivity to the SCWT, CPT and handgrip test between sessions (exercise and control) and times (pre and post). In case of significant interaction or main effects, the Tukey’s a *post-hoc* test was performed to perform the multiple comparisons. Paired *t*-tests were used to compare ambulatory BP parameters between session. For all tests, significance level was set at 5%. Continuous data are presented as mean ± standard deviation (SD) and categorical data are presented as relative frequencies. The minimum sample size required for the current study was calculated for changes in resting SBP (i.e. primary outcome), considering an alpha of 0.80 and effect size of 1.0 (SBP PEH net effect = −11 ± 11 mmHg) [21], which resulted in 13 participants.

## Results

### Participant characteristics

Forty RA women were invited to participate in the study and attended our laboratory to check full eligibility. Among them, 10 were not hypertensive and 10 did not agree to participate in the study after receiving detailed explanation about it. Therefore, 20 women with RA participated in this study (Table 1). By design, all participants were hypertensive and following different drug regimens, with angiotensin-converting enzyme inhibitor drugs (ACEi) and angiotensin receptor blockers (ARBs) being the most common antihypertensive medications. Participants also presented with a high prevalence of additional comorbidities and cardiometabolic risk factors, such as fibromyalgia (15%), osteoporosis (20%), depression (25%), obesity (45%) and dyslipidemia (35%). Table 2 presents the RA-related characteristics in the present study sample. Participants had a long disease duration (average of 14 years) and were distributed across different disease activity classes (i.e., from remission to high disease activity), and were under different anti-RA medications, with the more usual being prednisone and disease-modifying anti-rheumatic drugs (DMARDs).

**Table 1:** Participant characteristics.

	*n* = 20
Age (years)	52 ± 10
BMI (kg.m^−2^)	29.8 ± 6.0
Glucose (mg.dl^−1^)	96.55 ± 17.02
Total cholesterol (mg.dl^−1^)	178.45 ± 29.09
VLDL cholesterol (mg.dl^−1^)	23.21 ± 7.15
LDL cholesterol (mg.dl^−1^)	96.75 ± 21.65
HDL cholesterol (mg.dl^−1^)	52.00 ± 15.01
Triglycerides (mg.dl^−1^)	135.2 ± 64.84
hs-CRP (mg.l^−1^)	10.13 ± 19.07
ESR (mm.h^−1^)	13.94 ± 9.66
Smoking, *n* (%)	0 (0%)
Physical activity levels	
Active, *n* (%)	6 (30%)
Insufficiently active, *n* (%)	14 (70%)
**Comorbidities**	
Hypertension, *n* (%)	20 (100%)
Dyslipidaemia, *n* (%)	7 (35%)
Obesity, *n* (%)	9 (45%)
Hypothyroidism, *n* (%)	1 (5%)
Depression, *n* (%)	5 (25%)
Fibromyalgia, *n* (%)	3 (15%)
Lupus, *n* (%)	1 (5%)
Sjögren’s syndrome, *n* (%)	2 (10%)
Osteoporosis, *n* (%)	4 (20%)
Osteoarthritis, *n* (%)	2 (10%)
**Medications**	
Anti-hypertensive, *n* (%)	18 (90%)
ACEi, *n* (%)	2
ARBs, *n* (%)	9
Dihydropyridine calcium channel blockers, *n* (%)	2
ACEi + thiazides, *n* (%)	1
ARBs + thiazides, *n* (%)	1
ACEi + dihydropyridine calcium channel blockers + thiazides, *n* (%)	1
ARBs + dihydropyridine calcium channel blockers + thiazides, *n* (%)	2
Statin, *n* (%)	7 (35%)
Vitamin D, *n* (%)	11 (55%)
Calcium, *n* (%)	9 (45%)
Bisphosphonate *n* (%)	2 (10%)
Levothyroxine, *n* (%)	2 (10%)
Antidepressant, *n* (%)	5 (25%)
Proton pump inhibitor, *n* (%)	11 (55%)
**Hemodynamic variables and physical fitness**	
SBP (mm.Hg^−1^)	123 ± 16
DBP (mm.Hg^−1^)	81 ± 12
HR (beats.min^−1^)	80 ± 12
HR_peak_ (beats.min^−1^)	152 ± 21
VO_2peak_ (ml.kg^−1^.min^−1^)	18.7 ± 4.6

Continuous data are presented as mean±standard deviation, and categorical data are presented as counts and percentages.

*BMI* body mass index, *hs-CRP* high-sensitivity c-reactive protein, *ESR* erythrocyte sedimentation rate, *ACEi* angiotensin-converting enzyme inhibitors, *ARBs* angiotensin 2 receptor blockers, *SBP* systolic blood pressure, *DBP* diastolic blood pressure, *HR* heart rate, *VO*_2peak_ peak oxygen uptake during the cardiopulmonary exercise test.

**Table 2:** Rheumatoid arthritis parameters and treatment.

	*N* = 20
Disease duration (years)	14 ± 8
DAS28-CRP	2.94 ± 1.34
Remission, *n* (%)	9 (45%)
Low disease activity, *n* (%)	5 (25%)
Moderate disease activity, *n* (%)	4 (20%)
High disease activity, *n* (%)	2 (10%)
HAQ	1,0 ± 0.55
Pain-VAS (0–10 scale)	3.3 ± 2.1
**RA medications**	
Biological agents, *n* (%)	5 (55%)
DMARDs, *n* (%)	18 (90%)
Prednisone, *n* (%)	14 (70%)
NSAIDs, *n* (%)	6 (30%)
Muscle relaxant, *n* (%)	4 (20%)
Painkillers, *n* (%)	11 (55%)
Imunossupressants, *n* (%)	2 (10%)

Continuous data are presented as mean ± standard deviation, and categorical data are presented as counts and percentages.

*DAS28-CRP* disease activity score 28 with C reactive protein, *HAQ* health assessment questionnaire for rheumatoid arthritis, *DMARDs* disease modifying anti-rheumatic drugs, *NSAIDs* non-steroidal anti-inflammatory drugs (naproxen, celecoxib, meloxicam), Painkillers (acetaminophen, metamizole, tramadol, codeine); Muscle relaxants (cyclobenzaprine).

### Intervention

Mean HR during the exercise session was 99 ± 11 bpm, which corresponded to 64 ± 10% of HR_peak_. Mean HR during the control session was 81 ± 9 bpm, which corresponded to 52 ± 9% of HR_peak_.

### Main results

#### Rest

**Fig. 2:**
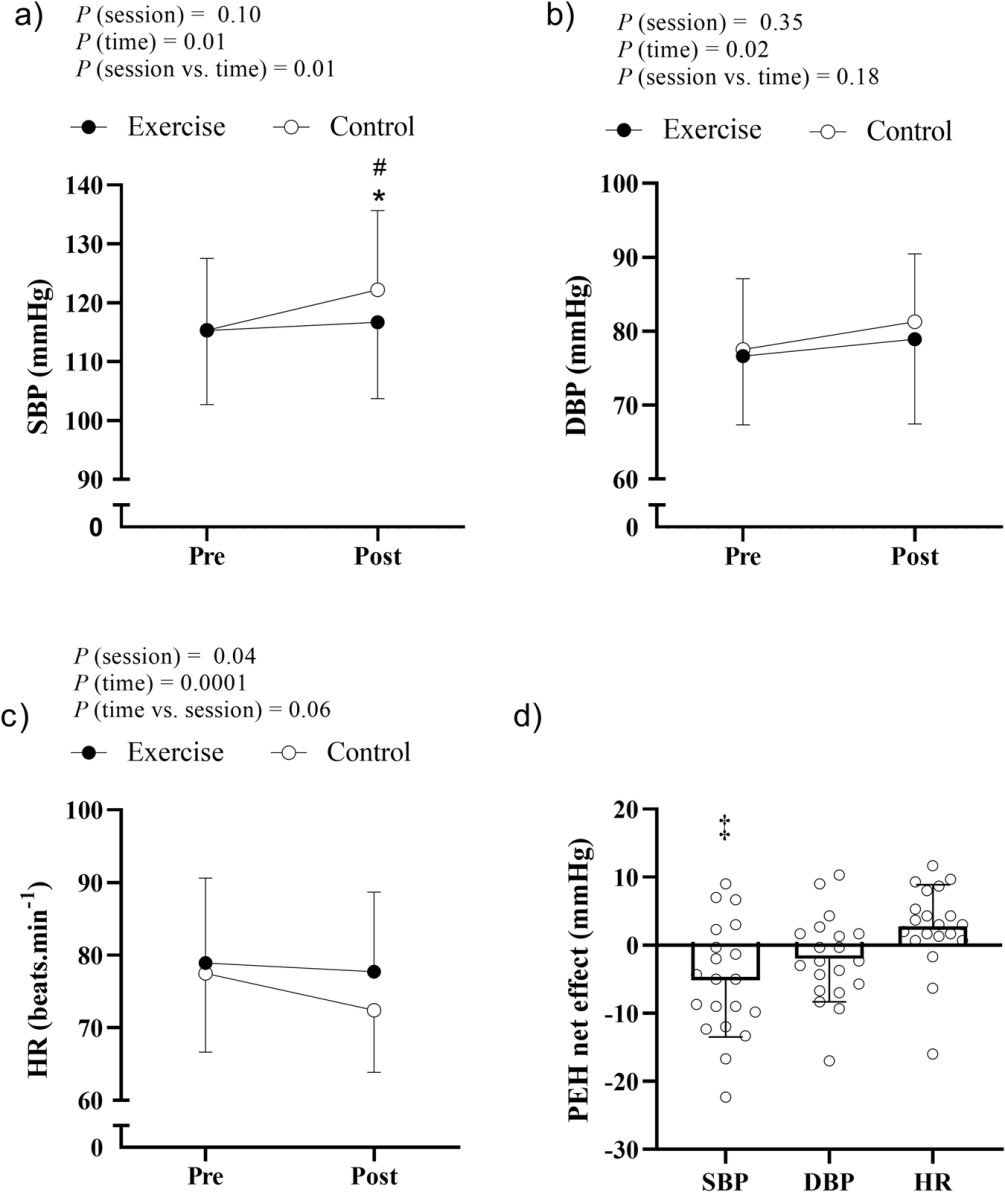
Resting blood pressure and heart rate before (pre) and after (post) exercise and control interventions. **a** SBP, systolic blood pressure; (**b**) DBP, diastolic blood pressure; (**c**) HR, heart rate. **d** post-exercise hypotension net-effect [PEH net effect = post-exercise BP – pre-exercise BP) – (post-control BP – pre-control BP)]. The white circles represent individual responses. A two-way (session vs. time) repeated measures analysis of variance was used to compare resting BP between sessions (exercise and control) and times (pre and post). In case of significant interaction or main effects, the Tukey’s a post-hoc test was performed to perform the multiple comparisons. PEH net effect was compared to zero using a paired Student’s *t*-test. #*P* < 0.05 post-control vs. post-exercise; *P < 0.05 post-control vs. pre-control, ‡ significant post-exercise hypotension (*P* < 0.05).

It was observed a significant interaction (*P* = 0.01) between session and time for resting SBP. There was no difference in resting SBP before and after exercise (*P* = 0.41), however resting SBP significantly increased from pre- to post-control (*P* < 0.01). Consequently, resting SBP was lower in the post-exercise compared with the post-control period (*P* < 0.01; Fig. 2a). Mean PEH net effect for SBP was −5 ± 8 mmHg (*P* = 0.01) (Fig. 2d).

On the other hand, there was no difference in responses of resting DBP and HR between sessions (*P* session vs. time = 0.18 and = 0.06, respectively; Fig. 2b, c). Regardless of the session, DBP was higher, and HR was lower at post compared to pre (main effect of time, *P* = 0.02 and *P* = 0.0001, respectively). Mean PEH net effect for DBP was −2 ± 6 mmHg (*P* = 0.18). Mean net effect for HR was +2 ± 6 mmHg (*P* = 0.06) (Fig. 2d).

#### Strop color and word test

**Fig. 3:**
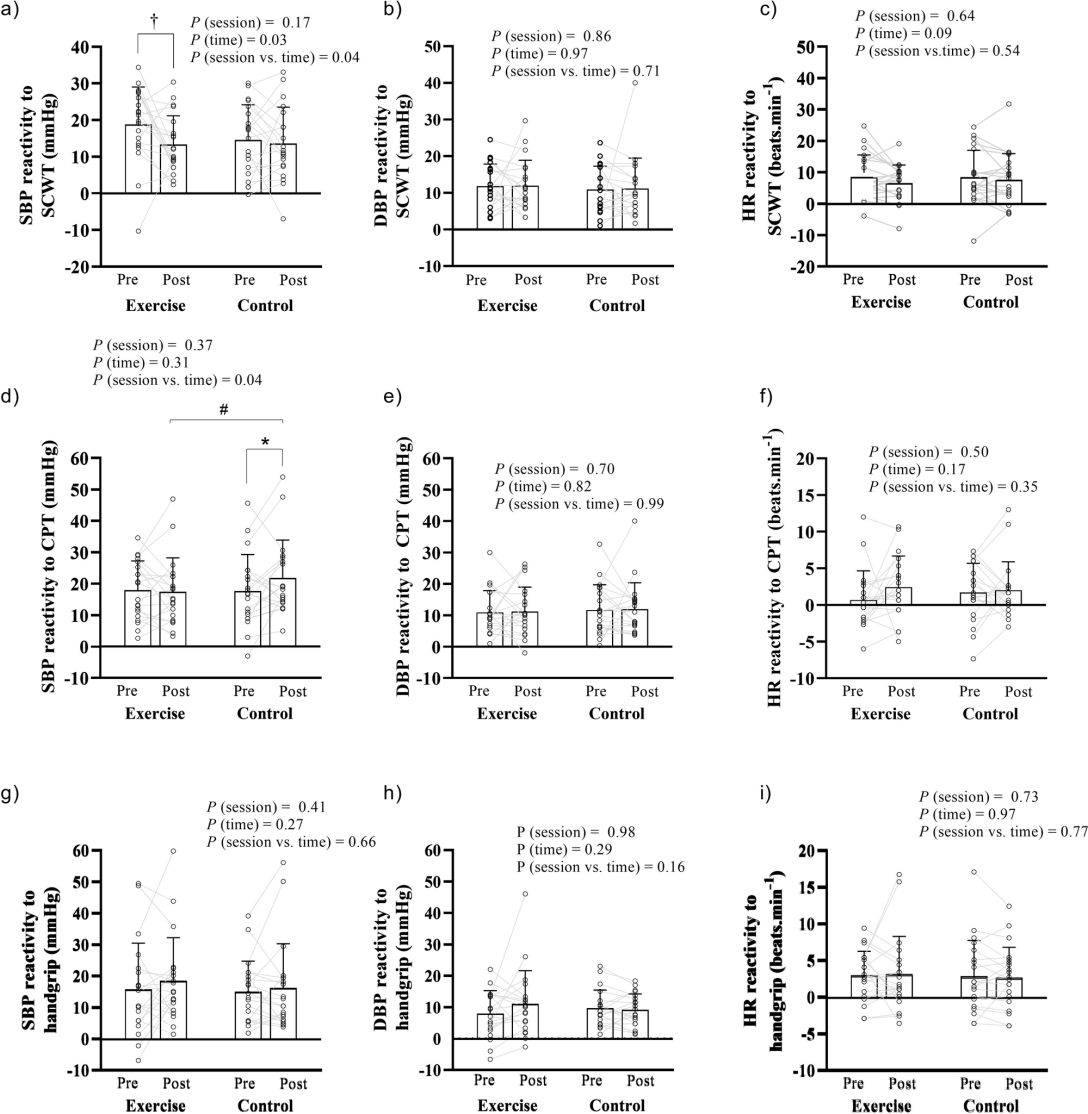
Blood pressure and heart rate reactivity to different stress tests before (pre) and after (post) exercise and control interventions. Systolic and diastolic blood pressure (SBP and DBP), and heart rate (HR) reactivity to the stroop color and word test (SCWT; panels **a**–**c**), cold pressor test (CPT; panels **d**–**f**) and handgrip test (panels **g**–**i**). Each line represents individual reactivity responses, and the bars present the mean reactivity responses (±standard deviation). A two-way (session vs. time) repeated measures analysis of variance was used to compare SBP, DBP and HR reactivity to the SCWT, CPT and isometric handgrip test between sessions (exercise and control) and times (pre and post). In case of significant interaction or main effects, the Tukey’s a post-hoc test was performed to perform the multiple comparisons. †*P* < 0.05 post-exercise vs. pre-exercise; *P < 0.05 post-control vs. pre-control; #*P* < 0.05 post-control vs. post-exercise.

It was observed a significant interaction (*P* = 0.04) between session and time for the SBP reactivity to the SCWT. SBP reactivity to the SCWT was lower after exercise compared to pre-exercise (−6 ± 9 mmHg; *P* = 0.04; the value in the bracket represents the net effect of the exercise session on SBP reactivity to SCWT, calculated as the difference of the SBP reactivity to SCWT between post-exercise and pre-exercise); on the other hand, there was no difference on SBP reactivity to the SCWT between pre- and post-control (0 ± 8 mmHg; *P* = 0.94; the value in the bracket represents the net effect of the control session on SBP reactivity to SCWT, calculated as the difference of the SBP reactivity to SCWT between post-control and pre-control), nor on SBP reactivity to the SCWT between post-exercise and post-control (*P* = 0.81; Fig. 3a). There were also no differences between DBP and HR reactivities to SCWT between sessions (*P* = 0.71 and *P* = 0.54, respectively; Fig. 3b, c).

There were no differences in the perceived stress during the SCWT between pre- and post-exercise (2 ± 1 vs. 2 ± 1, *P* = 0.14) and pre- and post-control (2 ± 1 vs. 2 ± 1, *P* = 0.26).

#### Cold pressor test

There was a significant interaction between session and time (*P* = 0.04) for the SBP reactivity to the CPT. SBP reactivity was not different between pre- and post-exercise (−1 ± 8 mmHg; *P* = 0.87; the value in the bracket represents the net effect of the exercise session on SBP reactivity to CPT, calculated as the difference of the SBP reactivity to CPT between post-exercise and pre-exercise), however it increased from pre- to post-control (+4 ± 10 mmHg; *P* = 0.04; the value in the bracket represents the net effect of the control session on SBP reactivity to CPT, calculated as the difference of the SBP reactivity to CPT between post-control and pre-control). Consequently, SBP reactivity to the CPT was lower on post-exercise compared to post-control (*P* = 0.04; Fig. 3d). On the other hand, there were no differences in DBP and HR reactivities to SCWT between sessions (*P* = 0.99 and 0.35, respectively; Fig. 3e, f).

There were no differences in the pain sensation during the CPT between pre- and post-exercise (6 ± 2 vs. 5 ± 3; *P* = 0.16) and pre- and post-control (6 ± 3 vs. 7 ± 4; *P* = 0.30).

#### Handgrip test

There were no differences in SBP, DBP and HR reactivities to the handgrip test between pre- and post-exercise and pre- and post- control (*P* = 0.99 and 0.35, respectively; Fig. 3g–i). There were also no differences in the pain sensation during the handgrip test between pre- and post-exercise (4 ± 2 vs. 4 ± 2; *P* = 0.53) and pre- and post-control (5 ± 3 vs. 5 ± 3; *P* = 0.57).

#### Ambulatory blood pressure

Two participants showed less than 80% valid ambulatory BP measurements after the control session; therefore, their data were excluded from the final analysis. Table 3 presents mean SBP, DBP and HR for the 24 h, awakening and asleep periods, as well as the nocturnal SBP and DBP dipping for the remaining 18 participants. There was no difference in any ambulatory BP variables between sessions.

**Table 3:** Ambulatory blood pressure in exercise and control sessions.

	Exercise	Control	*P*
24 h SBP (mmHg)	117 ± 10	117 ± 10	0.66
24 h DBP (mmHg)	75 ± 9	75 ± 10	0.87
24 h HR (beats.min^−1^)	79 ± 11	77 ± 9	0.73
Awake SBP (mmHg)	122 ± 11	120 ± 12	0.07
Awake DBP (mmHg)	81 ± 11	79 ± 11	0.28
Awake HR (beats.min^−1^)	84 ± 12	81 ± 11	0.31
Asleep SBP (mmHg)	108 ± 9	111 ± 10	0.33
Asleep DBP (mmHg)	66 ± 8	69 ± 8	0.13
Asleep HR asleep (beats.min^−1^)	71 ± 11	70 ± 9	0.68
SBP nocturnal fall (%)	11 ± 7	7 ± 6	0.10
DBP nocturnal fall (%)	14 ± 8	10 ± 6	0.07

Data are presented as mean ± standard deviation.

*SBP* systolic blood pressure, *DBP* diastolic blood pressure, *HR* heart rate.

## Discussion

This study explored the acute effects of aerobic exercise on resting BP, BP responses to multiple stressful stimuli, and 24-h BP in women with RA and hypertension. Overall, a single session of aerobic exercise resulted in net PEH for SBP and reduced the SBP response to the SCWT and CPT in this population.

This is the first study to assess the acute effects of exercise on BP in RA, a population with high prevalence and poor control rates of hypertension. In the present study, resting SBP remained stable from pre- to- post-exercise; however, it increased from pre- to post- control. The net decay in BP attributed to exercise accounted for the responses of control session was 5 mmHg, which represents a PEH magnitude similar to that observed in the general population [11], indicating that RA patients do not present impaired PEH. This is a clinically relevant decay in BP as reductions of 5 mmHg in SBP have been linked with significant reductions in the risks of mortality by stroke (−14%), coronary artery disease (−9%), and all causes (−7%) among hypertensive individuals [26]. Of relevance, acute reductions in BP across consecutive days of exercise are expected to accumulate and eventually lead to sustained reductions of BP over time [10], which may contribute to a better control of hypertension in RA.

In addition to reducing resting SBP, exercise also reduced SBP reactivity to two stressful tests. The SCWT is a neuropsychological test used to assess the ability to inhibit cognitive interference [27]. The mental stress elicited by this test also causes hemodynamic repercussions, which makes this test extensively used in studies assessing cardiovascular responses to stress [13]. In the present study, the SCWT caused increases of ~16 mmHg, ~12 mmHg, and ~8 bpm on SBP, DBP and HR, respectively. Interestingly, after exercise, SBP reactivity reduced by ~6 mmHg, which did not occur in the control session. This result indicates that a single session of aerobic exercise reduces SBP responses to mental stress conditions. It is well known that these situations may increase the risk of cardiovascular events (e.g., stroke and myocardial infarction) [28], therefore the reduction in SBP reactivity observed after exercise has the potential to reduce the risk of such cardiovascular events in RA.

The CPT evaluates the physiological responses to pain stimulation. In the present study, the CPT test caused increases of ~18 mmHg, ~11 mmHg, and ~1 bpm on SBP, DBP and HR, respectively. Additionally, 6 participants showed increase in SBP larger than 25 mmHg, which has been associated with increase in the risks of cardiovascular events and disease [29]. Interestingly, SBP reactivity to CPT decreased ~1 mmHg after exercise, whereas it increased ~4 mmHg after the control session, which culminates with a net reduction of ~5 mmHg in the SBP reactivity to this test. Besides reducing the risk of cardiovascular events [29], this attenuation of SBP reactivity to a pain-induction test is of relevance to RA patients as they may present with increased pain sensitivity and reduced pain threshold [30], and exposure to painful stimulation along their daily routine may induce significant cardiovascular responses leading to increased risks of cardiovascular events. It is also worth underscoring that 15% of the present study participants also had fibromyalgia, a long-term chronic pain condition, which further highlights the importance of exercise in reducing pain-induced cardiovascular responses.

This clinical study was not designed to elucidate the specific mechanisms behind the reduction in SBP at rest and in response to stress promoted by aerobic exercise. Acute exercise-induced hypotension may be caused by sustained vasodilation of the previously active muscle and reductions in centrally-mediated sympathetic activity or in sympathetic neurovascular transduction, [31]. Interestingly, patients with RA usually present impaired vasodilation following non-exercise stimulus (e.g., ischemia induced reactive hyperemia) [14]; however exercise is expected to upregulate vasodilatory mechanisms within the exercising muscle and in the systemic circulation [31] as consequence of hemodynamic stimulation and increased shear stress on vasculature [32]. Importantly, the reduction in SBP reactivity to SCWT and CPT cannot be attributed to reductions in the stress and pain caused by the test as participants reported them to be similar between pre- and post- exercise and control sessions. Future studies should investigate the effects of exercise on the vascular and neural regulation of BP in RA as this may improve the understanding of the mechanisms underlying the beneficial effects of exercise on BP in this population.

Contrary to the present study hypothesis, exercise did not reduce resting DBP, DBP reactivity to SCWT and CPT, and SBP and DBP reactivity to handgrip. We cannot tell why exercise did not promote reductions in DBP to the same extent as with SBP; however this data corroborates previous studies showing that a reduction in DBP after exercise usually occur to a lesser degree than in SBP [11, 20]. Specifically to the handgrip test, the absence of changes in SBP/DBP reactivity after aerobic exercise may be related to the high inflammatory levels of the participants as a previous study from our group has shown that hemodynamic responses to isometric exercise are positively associated with participant’s inflammatory burden [15]. Finally, ambulatory BP was also not different between sessions, which may be partially explained by the good control of the ambulatory BP in the present study participants. Indeed, contrary to our expectations and to previous data [8], mean values of 24 h, asleep and awake SBP/DBP were within normative values [25]. Use of multiple anti-hypertensive drugs and the optimized RA treatment may have helped the control of ambulatory BP, and potential additional effects of exercise were not possible to be detected (i.e., celling effect).

Results of the present study provide novel information that may contribute to the management of cardiovascular disease in RA. Hypertension has been reported as one of the strongest risk factors for cardiovascular disease in RA [5, 7], and rates of hypertension control in RA are significantly lower than in the general population [6, 8]. Use of medications, such as non-steroidal anti-inflammatory drugs and glucocorticoids may hamper the BP control in RA [33, 34], which reinforces the need for additional measures that may aid the control of hypertension in RA. In this scenario, the exercise-induced reductions in SBP in resting conditions and in response to two stressful stimuli observed in the present study highlight the importance of exercise in the management of cardiovascular risks in RA.

The present study is not without limitations. Firstly, the results of this study are limited to women with RA and multiple comorbidities and cannot be generalized to other RA subgroups. Secondly, the absence of measurements of local blood flow and vascular conductance, as well as of cardiac output and total peripheral resistance limits the understanding of the mechanisms behind the clinical responses observed in the present study. Additionally, participants’ physical activity level was assessed using a single question about their weekly amount of moderate-to-vigorous physical activity and recall bias might have affected these estimations. Another limitation was the potential carryover effect on BP across consecutive stressful tests, which may have affected the assessment of BP reactivity, specifically in the last test (i.e., handgrip test). However, 3–5 min interval between tests was employed to allow for the return of BP to baseline levels. Absence of a non-RA control group might also be considered a limitation as it prevents the assessment of the influence of RA on post-exercise BP. Finally, even though exercise promoted a PEH in SBP when compared to the responses observed in the control session, it should be noted that SBP did not decrease from pre- to post-exercise. In fact, SBP remained stable from pre- to post-exercise; therefore, the calculated PEH (i.e., 5 mmHg) was significantly influenced by the increase in SBP observed from pre-to post-control (~7 mmHg). It is not possible to draw conclusions as to why SBP increased in the control session, however prolonged time in sitting position [35] and repeated exposition to different stress tests may have played a role. This fact reinforces the importance of the present study ‘controlled’ design and the calculation of PEH considering the responses of both exercise and control sessions as previously discussed by our group [20]. However, based on the lack of reduction of SBP from post- to pre-exercise (when considering the exercise session alone), one may argue that the best phrasing for the present study results would be that exercise prevented an increase in SBP observed in the control session possibly related to the exposure to prolonged sitting and other factors (e.g., time of the day, body position, digestion, hydration levels, aspects related to the study procedures, etc).

In conclusion, a single session of aerobic exercise reduces resting SBP and SBP reactivity to mental stress and pain. These results reinforce the role of exercise in the management of cardiovascular disease in RA, via improving the BP control in this disease.

## Summary

### What is known about this topic

- Rheumatoid arthritis associates with higher rates and sub-optimal control of hypertension.

### What this study adds

- This study showed that a single session of exercise reduces resting systolic blood pressure (SBP) and SBP responses to stress in post-menopausal women with rheumatoid arthritis.
- These findings reinforce the role of exercise in the management of hypertension and cardiovascular disease in RA.

## Data Availability

The data that support the findings of this study are available from the corresponding author upon reasonable request.
